# Menstrual stigma and mental health for adolescent girls in South Sudan: A cross-sectional analysis

**DOI:** 10.1371/journal.pgph.0004916

**Published:** 2026-05-07

**Authors:** Ashley Smith, Alexandra Blackwell, Eleonora Mansi, Thomas Hussein, Ambaku Peter Lomena, Cosmas Ayella, Anywar Sam Okot, Kathryn Falb

**Affiliations:** 1 Ashley Smith – Department of Mental Health, Johns Hopkins Bloomberg School of Public Health, Baltimore, Maryland, United States of America; 2 Alexandra Blackwell– Department of International Health Johns Hopkins Bloomberg School of Public Health, Baltimore, Maryland United States of America; 3 Eleonora Mansi– International Rescue Committee, London, United Kingdom; 4 Thomas Hussein – International Rescue Committee, Juba, South Sudan; 5 Ambaku Peter Lomena– International Rescue Committee, Juba, South Sudan; 6 Cosmas Ayella– International Rescue Committee, Juba, South Sudan; 7 Anywar Sam Okot– International Rescue Committee, Juba, South Sudan; 8 Kathryn Falb – Department of International Health Johns Hopkins Bloomberg School of Public Health, Baltimore, Maryland, United States of America; Brock University, CANADA

## Abstract

Menstrual stigma, characterized by negative beliefs, attitudes, and practices surrounding menstruation, often leads to the social exclusion and discrimination of menstruators. The intersection of menstrual stigma and period poverty, which is marked by limited access to menstrual hygiene products and safe sanitation has a profound impact on the mental health and well-being of adolescent girls, particularly in low- and middle-income countries. This study aimedto examine the associations between menstrual stigma and mental health (Mood and Feelings Questionnaire) among female adolescents aged 10–20 in primary schools in Panyijar County, South Sudan. Cross-sectional baseline data were analyzed from a school safety evaluation using unadjusted and adjusted linear regression models In unadjusted analyses, knowing whether female classmates had their period was significantly associated with poorer mental health (β = 1.75, p < 0.001). Feeling ashamed if boys knew about one’s period was also associated with poorer mental health (β = 1.49, p = 0.05). In individually adjusted models, ajusting for demographic characteristics, feeling the need to hide one’s period was associated with better mental health (β = -1.80, p = 0.02). In fully adjusted models, which included all stigma variables and demographic controls, hiding one’s period remained significantly associated with mental health (β = -1.99, p = 0.04), while knowing when a classmate had their period showed a marginal association with poorer mental health (β = 0.88, p = 0.08). Disability was significantly associated with poorer mental health (β = 2.52, p < 0.01), while displacement showed a marginal association (β = 0.84, p = 0.06). No other demographic variables retained significance. These findings highlight the need for comprehensive strategies that address menstrual stigma and its impact on mental health, including interventions that challenge harmful social norms, promote safe latrine environments, and ensure access to hygiene products and menstrual education.

## Introduction

Puberty is a significant period of physical, emotional, and social transition, marking the time when a child experiences profound physical and hormonal changes, signaling the transition into adulthood [1]. Menstruation is a biological phenomenon, occurring monthly, that marks the onset of puberty for girls [2]. Around the world, menstrual experiences are shaped by gender and social norms [3]. In many cultures, the topic of menstruation is heavily stigmatized and considered taboo, perpetuating negative attitudes and misconceptions of menstrual health and hygiene [4]. In low- and middle-income countries (LMICs), the intersection between stigma and period poverty places girls experiencing menstruation at an increased risk for negative physical and mental health outcomes [5].

Menstrual stigma refers to negative belief, attitudes, taboos, and practices surrounding menstruation that leads to the marginalization of individuals experiencing periods [4]. This stigma manifests itself in different ways across cultures but is consistently linked with discrimination and social exclusion [4]. Oftentimes, girls who are on their period are seen as impure and dirty [3]. In many regions, girls who are on their menstrual cycles are often not allowed to participate in household chores, worship, school, or social activities [6]. The onset of menstruation and stigma associated with it also further perpetuates gender inequity. Girls who have gotten their menstrual cycle are also assaulted or harassed due to the linkages of menstruation with reproduction [7]. Aside from this, many societies view the onset of menstruation as the start of sexual maturity and is a sign girls are ready for early marriage that may raise risk for exploitation [6].

Period poverty, a global challenge, refers to the lack of adequate access to safe and hygienic menstrual products and education on reproductive health [8]. Despite over 1.9 billion individuals menstruating globally, a staggering rate of 500 million experience period poverty monthly [9]. Though period poverty is highly ubiquitous, it has a profound impact in LMICs. In resource poor settings, period poverty is often characterized by a lack of access to clean water, sanitation, and menstrual products, as well as knowledge deficits, limited social support, and emotional distress surrounding menstruation [5]. In many LMICs, access to safe water, sanitation, and menstrual products is minimal to none [10]. This lack of safe resources can pose serious health implications, such as reproductive and urinary tract infections as well as thrush [11]. Due to a lack of access to education on reproductive health, menstrual health literacy is also often limited and can result in unsafe menstrual hygiene practices as well as shame and isolation [7]. The impact of period poverty is particularly significant for adolescent girls. According to UNICEF, [12], half of schools in low-income countries lacked the adequate sanitation, water, and hygiene services necessary for girls experiencing menstruation. Research has shown that this lack of resources results in higher rates of school absenteeism, social isolation, and feelings of shame and embarrassment when trying to manage one’s menstrual cycle [13]. This further perpetuates gender inequality by undermining girls’ rights to education.

While menstrual stigma and period poverty are intersecting forces affecting girls across diverse contexts, their impact on mental health can vary significantly, with severity and contributing factors being shaped by social, cultural, and economic conditions. In many low- and middle-income countries contributing factors are linked to inadequate access to proper sanitation products or facilities and limited education on menstrual health and hygiene management [7]. For example, a growing body of literature suggests that in many low- and middle-income countries, due to a lack of education on menstrual hygiene, many girls are uninformed and unprepared for the onset of their menstrual cycle, leading to fear and anxiety about menarche [7]. Across LMIC’s menstrual hygiene management attributed to a lack of sanitary resources and clean water is a key concern [7]. As a result, various studies have reported that girl’s resort to utilizing old cloth blankets to manage their periods, and engage in discreet disposal of those materials out of shame or anxiety of being exposed to be menstruating [7]. In low- and middle-income regions such as Kenya, Uganda, and Zambia inadequate WASH infrastructures in schools have hindered girls’ abilities to manage their cycles with dignity and have increased rates of school absenteeism which in turn affects girls self-esteem and empowerment and reduces academic performance [7]. These factors intertwined with cultural and societal stigmas have a significant impact on the mental health and well-being of adolescent girls. Research has demonstrated that societal taboos surrounding menstruation often instill feelings of shame, embarrassment, isolation, and low self-esteem for menstruators [4]. A recent review conducted in 2023 found that individuals who experience period poverty, coupled with menstrual stigma, experience heightened levels of social isolation, stress, anxiety, and depression [11]. In conflict affected regions, although the contributing factors that shape the influence of period poverty and menstrual stigma on mental health are similar, they are often further compounded by a lack of resources, instability, and societal breakdown as well as deeply entrenched gender inequities and widespread violence [14]. Research has consistently shown that adolescent girls in conflict affected settings face higher rates of depression, anxiety, and trauma symptoms due to the compounded stress of violence, limited resources, and social exclusion [15].

One conflict affected context in which the intersection of period poverty and stigma and its effect on mental health is poignant is South Sudan.South Sudan gained its independence from Sudan on July 9, 2011 after decades of war and the loss of over 2 million lives [16]. Despite the people of South Sudan embracing hope for the future of the newly independent region, in 2013, two years after gaining independence, a large-scale civil conflict erupted due to political and ethnic tensions [17]. The conflict in South Sudan is still ongoing and as of 2023, 9.4 million individuals in the region are in need of humanitarian assistance [18].

Due to prolonged conflict, the social status of women and girls in the region is continuously deteriorating. These wars, compounded by high rates of alcoholism and the proliferation of small arms across the region, fuel physical and sexual violence, leaving adolescent girls—who make up 11% of South Sudan’s population—significantly vulnerable [6]. Against the backdrop of pervasive instability and socioeconomic upheaval, the issue of menstrual hygiene management is a pressing, yet often overlooked concern within the region. Conflict-related factors such as intermittent fighting, revenge killing, armed attacks, inadequate policing and rule of law, joblessness, recruitment of child soldiers, economic deficits, and displacement severely affect women and girls’ access to safe menstrual hygiene management [14]. Disruptions in supply chains hinder the availability of menstrual products, while fear of violence restricts mobility, preventing women and girls from seeking necessary support for menstrual hygiene management [19]. Research has shown that in conflict affected regions, due to existing gendered vulnerabilities, a lack of access to safe sanitation and menstrual products, leads to increased psychological distress and feelings of hopelessness, and anxiety [14]. Additionally, due to the conflict it is estimated that over 2 million individuals in South Sudan are internally displaced, with over 80% being women and girls, many of whom may reside in IDP camps [20]. Those residing in these camps face unique challenges in regards to sanitation such as a lack of access to safe latrines that are clean, lockable, and have adequate lighting [19].

Compounding factors influencing menstrual experiences include cultural taboos, discriminatory social norms, gender inequality, poverty, and a lack of basic sanitary services.In the region, menstruation is often referred to as *Ada shaharia* or the usual monthly [21]. This phrase underscores the pervasive silence and stigma surrounding menstruation in the country [21]. In a 2014 study conducted on young femaleson perceptions surrounding menstruation across South Sudan, 58.2% of participants believed that menstruation made them unclean, 28.4% reported that they considered menstruation a disease, and 48.4% believed it to be dangerous [22]. Research has shown that in many areas, adolescent girls on their cycle are forbidden from cooking, going to school, and going to church [2]. They are also advised to stay away from boys [2]. In regions where girls are allowed into society during menstruation, they are often shamed or harassed if they are discovered to be menstruating [2]. The fear of shame and harassment compels girls to remain silent about their periods, making it challenging to address their needs in South Sudan.

Across the region, many girls seek guidance on menstruation from their mothers [2]. However, due to its taboo nature, information on safe and hygienic practices for menstrual cycles are unknown or discussions are avoided entirely [16]. Overall, it is estimated that at least 66% of adolescent girls have little knowledge or incorrect knowledge of menstrual hygiene and many had no knowledge of menstruation before its onset [23]. Management of menstrual cycles is further compounded by a lack of safe water, sanitation, and hygiene products. Many individuals use rags, cartons, leaves, and other dirty materials to manage their cycles due to how expensive and inaccessible sanitary pads are [23].

Little research has been conducted on the associations between menstrual stigma and mental health for adolescent girls in South Sudan. This study aims to fill a critical gap in understanding the relationship between menstrual stigma and mental health to provide a foundation for targeted interventions and policy recommendations to improve sanitary conditions and mental health for girls in South Sudan. Grounded in the socio-ecological framework, this study considers the layered influence of individual, interpersonal, organizational, and structural factors on adolescent’s menstrual experiences and associated mental health outcomes. It posits that stigma associated with menstruation operates through multiple channels across the social ecological framework including individual beliefs, community norms, and structural conditions-ultimately influencing adolescent girls’ mental health. A recent scoping review highlighted that factors associated with menstruation—including stigma, lack of information, limited access to menstrual products, and inadequate menstrual management facilities—are associated with both physical and emotional health outcomes, including anxiety, distress, and depression [24]. Studies have shown that at the individual level, internalized stigma may shape girls’ beliefs about menstruation and self-worth [4]. Interpersonally, family and peer attitudes may reinforce shame and silence [2]. Organizational influences, such as inadequate sanitation infrastructure, may limit girls’ ability to manage menstruation with dignity [2]. Finally, structural factors—including gender norms, poverty, and displacement—create broader constraints on menstrual health management and psychosocial well-being [14]. By applying this framework, the study examines how menstrual stigma and its multi-leveled influences contribute to mental health challenges among adolescent girls aged 10–20 in primary school in Panyijar County, South Sudan.

## Methodology

### Study design

This study is a secondary analysis of de-identified baseline data collected between April - June 2021 by the International Rescue Committee (IRC) as part of a quasi-experimental study to evaluate the effectiveness of a school safety intervention [25]. Fifty-two schools in the towns of Nyal and Ganyiel in Panyijar County were eligible for participation and recruited to participate in the study. Additional study protocol details have been published elsewhere [25].

### Study population

Participants included 1272 respondents across 32 primary schools in the greater Nyal and Ganyiel located in Panyijar County in South Sudan. Participants were students ages 10–20 original study. Participants were randomly selected school-enrolled adolescents aged 10–20 years, reflecting the full age range of adolescents enrolled in schools within the target population at the time of data collection. Experiences and perceptions of menstrual stigma differ across ages and to account for this, age was included as a covariate in all analytic models. This approach allowed us to retain the full school-enrolled adolescent population while adjusting for age-related differences in experiences of menstrual stigma.The present secondary analysis was restricted to female participants given they were asked questions related to menstruation (N = 277).

### Ethics statement

This study is a secondary analysis of de-identified data originally collected by the International Rescue Committee. The original study protocols and tools were reviewed and approved by the IRC Institutional Review Board (CYPD 1.00.015) and a local Technical Advisory Group consisting of representatives from local authorities in Panyijiar County and humanitarian agencies working in schools.

Data was collected in accordance with global ethical standards for studies involving children and research on violence and was approved by the IRC ethical review board (CYPD 1.00.015). Interviews with participants were conducted in private and secure locations to ensure confidentiality [25]. Consent was first sought from the schools and teachers for participation in the research. Secondly, the program team administered sensitization activities to introduce the study and program to parents and guardians. Parental consent and informed oral assent was sought for children ages 10–17 and informed oral consent was sought for adolescents 18–20. All participants were provided with referral information for follow-up services. The secondary analysis was reviewed and approved by the Institutional Review Board (IRB) at Johns Hopkins Bloomberg School of Public Health (Reference Number: 00027453).

### Data collection and measures

The measures for data collection were originally developed in English and translated verbally to the Neur language. and underwent linguistic and cultural adaptation for the South Sudanese context, including translation into Nuer by child protection and Monitoring, Evaluation, Accountability, and Learning (MEAL) staff fluent in both English and the local language [25]. The translated versions were reviewed during enumerator trainings to ensure clarity, cultural relevance, and conceptual equivalence and was subsequently back-translated into English to assess consistency and accuracy of meaning [25].

Data collection was conducted by trained enumerators who delivered the questionnaires verbally, and then recorded the participants’ responses into a tablet using Kobo Collect [25]. We conducted a demographic questionnaire and obtained variables such as gender, age, disability level, grade, displacement status, and study site [25]. Menstrual stigma was measured continuously using an adapted version of a questionnaire on period stigma for boys and girls developed and validated in Tanzania [26]. In Tanzania, the questionnaire was reviewed by research assistants from a local university to ensure clarity and cultural appropriateness, and the survey was additionally pilot-tested in a U.S. population prior to data collection [26]. Mental health was measured continuously through an adapted version of the Mood and Feelings Questionnaire (MFQ) [27], for which a higher score indicates poorer mental health (Range: 0–24; Cronbach’s alpha = 0.88).

### Data analysis

Descriptive statistics were generated to assess the correlation between demographics and outcomes of interest. A robustregression model accounting for clustering at the school level was used to analyze the relationship between the dependent variable of mental health and independent variables of menstrual stigma and demographic characteristics. All analyses were conducted with STATA statistical software version 18 Basic Edition [28] and the secondary analyses were completed between 10/01/2024 – 01/05/2024.

## Results

### Descriptive statistics

On average, participants were 16.4 years of age (SD = 1.6) and were in grade 5 (SD = 2.2). 41.2% were at the Ganyiel school site and 58.8% were at the Nyal site (Table 1). The majority of participants reported having no disability with regarding to functional difficulty (73.6%). On average, 46.7% of participants had experienced displacement. Overall, the mean score of the MFQ was 4.2 (sd = 4.3).

**Table 1:** Descriptive statistics of the study sample at baseline. (N = 277).

	Mean (SD) % (N)
**Demographics**	
**Age**	16.4 (1.6)
**Grade**	5.2 (2.2)
**Site**	
Ganyiel	41.2% (114)
Nyal	58.8% (163)
**Disability Level**	
None	73.6% (204)
Mild	15.9% (44)
Moderate/Severe	10.4% (29)
**Displacement Status**	
Yes	46.7% (129)
No	53.3% (147)
**Mental Health**	4.2 (4.3)
**Menstrual Stigma**	
Are you able to go to school when you have your period?	
Yes	48.4% (134)
No	50.9%(141)
Do you feel safe in the latrines at school when you have your period?	
Yes	36.8% (102)
No	61.0% (169)
Do you know when your female classmates have their period?	
Yes	33.6% (93)
No	65.0% (180)
Do you feel like having your period is something that one must hide from others?	
Yes	73.6% (204)
No	23.8% (66)
Would you feel ashamed if other girls knew that you were on your period?	
Yes	62.5% (173)
No	36.1% (100)
Would you feel ashamed if boys knew that you were on your period?	
Yes	91.3% (253)
No	7.2% (20)
If people knew that you have ever had your period, would you be afraid of teachers not being understanding or helpful?	
Yes	48.4% (134)
No	46.6% (129)
Do you feel ashamed during your period?	
Agree	8.7% (24)
Disagree	20.2% (56)
Neutral	70.0% (194)

Table 1 demonstrates frequencies of items related to menstrual stigma. Less than half of participants (48.4%) felt they could go to school during their period and approximately one-third (36.8%) felt safe in latrines at school when on their period. One-third of girls (33.6%) reported knowing when a female classmate has their period. Nearly three out of four girls (73.6%) reported feeling like a period is something one must hide, and the majority also reported feeling ashamed if other girls knew they were on their period (62.5%). Stigma was more pronounced in instances when boys might know if they are on their period, with over nine out of ten girls (91.3%) indicating they would be ashamed. Approximately half of girls (48.4%) felt teachers would not be understanding or helpful if they knew they were on their period.

### Associations between menstrual stigma and mental health

In unadjusted linear regression accounting for clustering at the site level (Table 2), knowing whether female classmates had their period is the had a statistically significant association with mental health (β = 1.75, p < 0.001; 95% CI:0.39, 3.11). Additionally, feeling ashamed if boys knew you were on your period had a statistically significant association with poorer mental health (β = 1.49, p = 0.05; 95% CI: -0.02, 3.01).The individually adjusted column in Table 2 presents separate models for each menstrual stigma item, controlling for individual demographic characteristics such as respondent age, grade in school, disability, and displacement, while the fully adjusted column combines all menstrual stigma items into a single model, accounting for the other stigma items and controlling for individual demographics. Both accounted for clustering at the site level. In the individually adjusted model, feeling the need to hide one’s period was associated with better mental health in the individually adjusted model (β = -1.80, P = 0.02; 95% CI: -3.26,-0.35). In the fully adjusted model, feeling like you must hide your period from had a statistically significant association with mental health (β = -1.99 (0.04; -3.86, -0.13) while knowing when a female classmate had their period trended toward astatistically significant association with poorer mental health (β = 0.88, p = 0.08; 95% CI:-0.14, 1.89). Moreover, disability (β = 2.52 p < 0.01; 95% CI: 1.67, 3.37) was statistically significant, while displacement (β = 0.84,p = 0.06; 95% CI: -0.18, 1.62) was partially significant when controlled for in the fully adjusted model, suggesting that these demographic factors have an important role in mental health outcomes. No other control variables retained significance.

**Table 2:** Unadjusted association between menstrual stigma and mental health (N = 277).

	Unadjusted Model	Individually Adjusted Model^i^	Fully Adjusted Model^ii^
Menstrual Stigma Item	β (p-value; 95% CI)	β (p-value; 95% CI)	β (p-value; 95% CI)
Are you able to go to school when you have your period?	-0.24 (0.82; -2.33, 1.86)	-0.17 (0.77; -1.37, 1.02)	-0.21 (0.67; -1.23,0.81)
Do you feel safe in the latrines at school when you have your period?	0.01 (0.99; -1.89, 1.89)	-0.36 (0.60; -1.78, 1.06)	-0.58 (0.42; -2.03, 0.88)
Do you know when your female classmates have their period?	1.75 (0.01; 0.39, 3.11)	0.68 (0.29; -0.59, 1.94)	0.88 (0.09, -0.13, 1.89)
Do you feel like having your period is something you must hide from others?	-2.46 (0.06;-5.06, 0.14)	-1.80 (0.02; -3.26, -0.35)	-1.99 (0.04; -3.86, -0.13)
Would you feel ashamed if other girls knew that you were on your period?	-0.45 (0.53, -1.92, 1.02)	-0.18 (0.14;-1.92, 0.29)	-0.76 (0.19; -1.93, 0.41)
Would you feel ashamed if boys knew that you were on your period?	1.49 (0.05; -0.02,3.01)	-0.09 (0.86; -1.23, 1.03)	1.07 (0.47, -1.91, 4.04)
If people knew that you have ever had your period, would you be afraid of teachers not being understanding or helpful?	-0.29 (0.75; -2.12, 1.55)	-0.04 (0.93;-1.08, 0.99)	-0.06 (0.97; -1.22,1.27)
Do you feel ashamed during your period?			
Reference	1	1	1
Neutral	-0.92 (0.43; -3.26, 1.41)	-0.55 (0.57; -2.49, 1.39)	-0.69 (0.35;-2.20,0.81)
Agree	0.74 (0.47; -1.31, 2.80)	0.35 (0.68; -1.35, 2.04)	0.07 (0.97; -1.22, 1.23)

^i^ Adjusted for individual demographic characteristics (age, grade, disability level, and displacement status).

^ii^ Adjusted for all other menstrual stigma items and individual demographic characteristics.

## Discussion

Menstrual stigma is a pervasive and deeply entrenched issue faced by millions of girls and women worldwide. In areas with limited resources, such as South Sudan, menstrual stigma is exacerbated by existing challenges related to access to clean water, sanitation, and lack of menstrual hygiene products and education [4]. The findings of this study demonstrated significant associations between various dimensions of menstrual stigma and poor mental health outcomes for adolescent girls in South Sudan.

One of the key findings of this study was a significant association between boys knowing when a girl is on her period and poorer mental health, which aligns with existing literature on menstrual stigma and gendered power dynamics. One reason why it may not reflect statistical significance is that while stigma from male peers may be emotionally distressing, in the cultural context of South Sudan where menstrual stigma is deeply rooted in gendered and sociocultural norms, girls may underreport the extent of which they feel boys are aware or judging them on their periods due the normalization of such stigma in the region. Cultural norms dictate that menstruation is a taboo topic that should be kept silent and that girls who are menstruating should avoid interactions with those of the male gender. These norms stem from deeply ingrained beliefs that those who are menstruating are no longer considered modest but are instead “unclean and impure” [3]. This gendered norm contributes to the isolation of girls who are on their period which negatively impacts mental health and may also normalize bullying and harassment from males when menstruation is discovered. In a study conducted in 2024 on the experiences of adolescent girls, participant reported being shamed or bullied by male peers if menstrual blood becomes visible on their clothing [2]. Another study found that found that 52.6% of girls in South Sudan reported being laughed at by boys during menstruation, with 35.2% experiencing abuse and 21.3% subjected to name-calling [23]. The psychological impact of menstrual stigma coupled with gendered expectations of privacy, could contribute to a heightened sense of anxiety, low self-esteem, or depression for girls in the region if menstruation is discovered by males [2].

Additionally, a significant association was found between knowing when others have their periods and poor mental health. Previous research has shown that inadequate sanitation facilities and lack of privacy during menstruation can heighten feelings of exposure, shame, and anxiety, which in turn negatively impact mental health [3,29]. In South Sudan, where it is estimated that roughly 80% of schools lack proper sanitation facilities for menstrual hygiene management, it is difficult for adolescent girls to manage their periods safely and with dignity [30]. Privacy is often limited and may increase the likelihood that girls will become aware of other’s menstrual cycles. This knowledge could heighten concerns of one’s own menstruation being noticed or discovered by others potentially invoking internalized stigma surrounding menstruation and feelings of shame, embarrassment, isolation, and anxiety [4].

Our study also identified that feeling the need to hide one’s period was significantly associated with greater mental health. This finding is counterintuitive and should be interpreted with caution. In a setting like South Sudan where menstrual stigma is pervasive, concealment may function as a contextually adapted coping strategy that enables individuals to navigate societal expectations and avoid stigma-related stressors, rather than being inherently beneficial for mental health. The literature has shown that in many areas across the region, adolescent girls on their cycle are forbidden from cooking, going to school, and going to church while on their period [2]. They are also advised to stay away from boys [2]. In regions where girls are allowed into society during menstruation, they are often shamed or harassed if they are discovered to be menstruating [2]. Additionally, the onset of menstruation is often linked to readiness for marriage, subjecting girls to early and forced marriages [31]. In this context, concealing one’s menstrual cycle can be a means to maintain personal agency and protect against the social and psychological consequences of menstrual stigma and mental health. Further research is needed to explore the complex relationship between menstrual stigma, mental health, and coping strategies in such contexts, and to inform more sustainable, culturally sensitive approaches to addressing menstruation and associated psychological well-being.

Although latrine safety and school absenteeism were not statistically significant in our final models, these variables remain critical areas of concern that warrant further investigation. The lack of significance may be attributable to limitations in sample size or unmeasured confounding variables, rather than a true absence of effect. It is also possible that those most affected—such as girls who are chronically absent from school due to menstrual stigma or safety concerns—were not captured in the sample, leading to an underestimation of these associations. In general, sanitary resources across South Sudan, are inadequate, and one area adolescent girls are particularly impacted in is education. Schools in South Sudan often lack proper sanitation facilities, infrastructure, and privacy to meet the menstrual hygiene needs of adolescent girls [21]. Due to this as well as fears of public shaming, it is estimated that girls experiencing their menstrual cycle in the region are expected to miss 10–20% of school days managing their periods [32]. The deficiency of safe resources and sanitation facilities across the region poses not only significant physical health risks, but also impacts mental health [11]. Literature has demonstrated that girls who do not have access to safe sanitation resources or facilities are at an elevated risk for experiencing anxiety, depression, and psychological distress [29]. For example, due to the taboo nature of menstruation in South Sudan, those who are on their periods may have anxiety or fear over subjection to shaming and harassment if they are discovered if they are on their periods if there is a lack of privacy in sanitation facilities or if they don’t have access to materials that can prevent menstrual leakages and accidents [2].

One possible reason for the lack of statistical significance for school absenteeism is that the data may not fully capture the experiences of girls who are already disengaged from school due to chronic stigma or other barriers—meaning those most affected may be missing from the sample. The result regarding school absenteeism reflects current literature suggesting menstrual stigma and related school absenteeism significantly impact adolescent girls’ mental health, empowerment, and academic achievement [2]. In the context of South Sudan, school absenteeism due to menstruation may reflect societal beliefs and structural barriers. In the region, girls who are menstruating often experience social exclusion and restrictions on daily activities, such as attending school which may lead to feelings of shame, isolation, and low self-esteem [6]]. In addition, according to the General Education Strategic Plan 2017–2022, 20% of children in South Sudan reported that their path to school felt unsafe, and over half stated that they did not feel safe using the school latrines [21]. For girls, these insecurities are acute. Sociopolitical instability and conflict has heightened the threat of sexual violence, abduction, and harassment by armed actors is prominent on school routes [33]. In the region, girls do not have access proper sanitation products and may fear the prospect of bleeding through their clothing or being discovered to be on their period and subjected to stigma-related harassment, assault, or embarrassment on an already unsafe path to school. This combination of physical insecurity and menstrual stigma may lead to school absenteeism as a form of self-protection and may further perpetuate anxiety and stress. The lack of proper sanitation facilities in schools for managing menstruation also affects school attendance rates. For example, in a study conducted in South Sudan in 2021, it was found that limited access to hygiene products, inadequate sanitation facilities, and stigma surrounding menstruation contributed to lower rates of academic achievement and attendance at school [2]. Without access to clean, private, and safe latrines or sufficient menstrual hygiene products, many girls may choose to stay home rather than risk the psychological burdens of shame and embarrassment if they are discovered to be on their menstrual cycle. Together, these challenges underscore how both environmental and psychosocial factors intersect to undermine adolescent girls’ physical and emotional well-being and development.

The findings within this study highlight the need for humanitarian mental health and psychosocial programming to consider dimensions of menstrual stigma for adolescent girls as an important pathway for change. In one perspective, community-based interventions may be developed to target the harmful social norms and taboos surrounding menstruation in the region. This could begin with sensitization efforts and workshops that provide education on menstruation and hygiene management as well as work to explore the role of gendered power dynamics in relation to beliefs around menstruation and how this impacts adolescent girls’ physical and emotional well-being. Trusted community members could be trained to model more supportive attitudes towards menstruation and solidarity with females experiencing their cycle. Rather than immediately initiating open dialogue regarding menstruation, due to its taboo nature, early efforts may prioritize building trust, quiet reflection, and informal peer support networks for adolescent girls that lay the groundwork for more open conversations on the topic as community readiness grows. Over time safe and empowering spaces can grow where adolescent girls can be empowered to talk about their menstrual cycle and hygiene management freely. Additionally, polices and intervention approaches targeting the menstrual education needs of adolescent girls, creation and utilization of safe hygiene products, and the development of safe latrine spaces are essential components for addressing menstruation needs and stigma in South Sudan. Interventions centered around construction of sanitation infrastructure, awareness raising activities and trainings on menstrual health and hygiene, open communication on menstruation, and the creation and distribution of sanitary pads have been conducted globally in regions such as Ghana Nepal, Ethiopia, and Madagascar and have proven effective in increasing menstrual knowledge and improving physical and psychosocial outcomes related to menstruation [34]. In a study conducted on the effects of WASH sanitation infrastructure, menstrual products, and teacher awareness intervention for adolescent girls in Madagascar, results showed decreased anxiety, higher menstrual hygiene knowledge, and engagement in improved menstrual hygiene management behaviors [35].

While this secondary data analysis contributes to the literature on menstrual stigma and mental health, limitations that should be considered when interpreting these results. Firstly, the measures used within this study relied on self-reporting and were conducted within or near schools leaving the results susceptible to social desirability bias. Despite confidentiality measures being in place, given the sensitive nature of experiences related to menstrual stigma and menstruation being such a taboo topic in the region, participants may have felt uncomfortable disclosing their true experiences of menstrual stigma and may have opted to select responses that they found more socially acceptable. This could have led to underreporting of menstrual stigma within the study. Another limitation within the study is sampling bias. The literature has shown that some girls within South Sudan may drop out of school due to their periods [36]. From a more generalized perspective, in the Unity State, drop-out rates may be higher due to instability, ongoing conflict, unsafe school paths, discrimination, and high rates of gender-based violence [37]. This sampling bias may have led to an underrepresentation of students who have experienced menstrual stigma leading to an incomplete understanding of this type of stigma within the sample. Additionally, while the MFQ is a validated tool for assessing mental health symptoms in adolescents, it was developed in Western contexts and has not been culturally adapted or validated for the South Sudanese context. The MFQ, although a standardized tool, was developed to reflect Western conceptualizations of mental health and lacks cultural grounding. Without adaptation to the local setting, there is a risk for category error- the misinterpretation or under-detection of local expressions of distress and misclassification of culturally normative behaviors as pathological [38]. Similar concerns apply to the menstrual stigma questionnaire which was adapted from a questionnaire utilized in Tanzania but not further re-validated in the South Sudanese context. While this measure captures key domains of menstrual stigma found in other research studies,the cultural nuances, meanings, social consequences, and manifestation vary across cultures. Without construct validation, there is a risk that the measure may not have fully captured local culturally relevant forms of stigma and its output. It is important to note that, South Sudan’s sociopolitical context, influenced by its internal conflict and displacement, creates a context in which the manifestation of stigma and distress is shaped by prolonged exposure to violence, loss, and instability [14]. This complexity may further hinder accurate assessments of mental health and when using Westernized tools developed in contexts less influenced by conflict. Future research should center on adapting and validating mental health assessment tools to reflect local expressions of distress and the sociopolitical-driven realities of the context and their influence on these idioms. In addition, ongoing conflict likely exacerbates stress and negatively impacts the mental health of adolescent girls. Consequently, the observed associations between menstrual stigma and mental health may be influenced by the broader context of conflict and instability. Future research should also examine the relationship between menstrual stigma and mental in less conflict-affected low- and middle-income countries (LMICs) to better understand the impact in settings with lower levels of context-related stress.

## Conclusion

In conclusion, this secondary analysis contributes to the limited literature on the associations between menstrual stigma and mental health for adolescent girls. Notably, we found significant relationships between knowing other girls have their periods, feeling shame about periods, andfeeling ashamed if boys knew you were on your period. It is also important to note that although other factors such as latrine safety and school absenteeism associated with menstrual stigma within the study were not proven to be significant, they still provide valuable insight into the complex nature of menstrual stigma. These findings underscore the need for comprehensive approaches to address menstrual stigma and its associations with mental health including targeted interventions that address harmful social norms surrounding menstruation and promoting safe latrine spaces, hygiene products, and education about menstruation.
